# When fluency outpaces provenance: source traceability and digital health literacy against AI health misinformation

**DOI:** 10.3389/fpubh.2026.1986759

**Published:** 2026-10-20

**Authors:** Mingsheng Peng, Lu Feng

**Affiliations:** School of Culture and Creativity, Beijing Normal–Hong Kong Baptist University (BNBU), Zhuhai, China

**Keywords:** calibrated trust, digital health literacy, generative artificial intelligence, health literacy, health misinformation, infodemic management, provenance, source traceability

## Abstract

Generative artificial intelligence (AI) can make health explanations accessible while separating persuasive language from inspectable evidence. This creates a public health problem beyond plainly false content: an answer may mislead through unsupported attribution, obsolete evidence, omitted uncertainty, or inappropriate application. This Conceptual Analysis uses a targeted integrative approach to connect health literacy, misinformation, trust, large language model evaluation, and content provenance around the pathway from a generated claim to a health decision. Its contribution is an integration and operationalization of established concepts, not a new literacy construct or truth-certification mechanism. We distinguish five source traceability functions: origin, claim, evidentiary, procedural, and correction traceability. These functions interact with four iterative, context-dependent user practices: inspect, triangulate, calibrate, and escalate. Calibrated trust means discriminating between stronger and weaker support and relying proportionately on it, rather than maximizing or minimizing trust. Effective traceability also requires accessible, comprehensible evidence pathways and reasonable verification effort. Institutional and platform accountability is therefore part of the model, not an optional supplement to individual competence. We provide illustrative operational indicators, provisional risk-tiered safeguards, and six testable propositions organized around evidentiary discrimination, confidence calibration, decision appropriateness, verification behavior, verification burden, and escalation behavior. Neither the unified model nor its indicators or risk tiers have been empirically validated. The framework offers a decision-oriented research and design architecture for evaluating when evidence visibility and literacy can jointly support safer use of synthetic health information.

## 1 Introduction

Digital health communication has entered a synthetic phase. People no longer encounter health information only through identifiable webpages, institutions, professionals, or social media accounts. They increasingly receive newly generated answers that combine explanation, recommendation, summary, and citation in a conversational interface. This shift can widen access to plain-language information and support health information seeking (1, 2). It can also make unsupported statements appear coherent, responsive, and personally relevant. The public health concern is therefore not limited to the volume or speed of misinformation. It also concerns the weakening of the visible connection between a health claim and the evidence, institution, or accountable actor behind it.

Synthetic health information is used as a neutral term for generated content. AI health misinformation refers here to false or materially misleading health information. Material misleadingness includes an inaccurate basis for judgment created through unsupported attribution, obsolete evidence, fabricated or misaligned citations, omitted uncertainty, or contextually inappropriate application. Evidentiary inadequacy and contextual inappropriateness are distinguished from factual falsity, and not every incomplete or uncertain output constitutes misinformation. Intent to deceive is not presumed; deliberate deceptive production is termed disinformation where established.

Health misinformation can influence decisions, erode institutional trust, and impede public health responses (3, 4). False information travels effectively in networked environments, and exposure can alter consequential health intentions (5, 6). Generative AI adds a distinctive mechanism: plausible text can be synthesized without preserving a stable route from each claim to a reliable source. Earlier evaluations document useful responses alongside incomplete, guideline-inconsistent, or misleading material (7–9). Bibliographic audits also identify fabricated or inaccurate references (10–12). More recent research separates citation validity from statement-level support (13), and audits accuracy and referencing under adversarial prompts (14). These studies document specific failure modes, not population-wide error rates for all current systems.

Health literacy and digital health literacy are central resources for navigating this environment. Health literacy includes the capacities to access, understand, appraise, and apply health information (15). eHealth and digital health literacy extend these capacities to networked information, interactive tools, and changing technological settings (16, 17). These concepts correctly emphasize that comprehension alone is insufficient. Users also need appraisal, navigation, communication, and application skills. However, a focus on individual competence can become inadequate when systems obscure how outputs were generated, fail to map claims to sources, or present unverifiable citations. No amount of user skill can inspect evidence that an interface does not expose.

Recent scholarship addresses AI health literacy, consumer-facing model evaluation, misinformation susceptibility, and AI-driven epistemic disruption (18–21). A further AI health literacy framework links patient, clinician, and governance competencies, including trust calibration and critical appraisal (22). These approaches already recognize verifiability, contextual judgment, or institutional responsibility. The contribution here is not to invent health, digital/eHealth, or AI health literacy, provenance, transparency, verification, susceptibility, or accountability. It is to integrate and operationalize these concepts around the decision pathway from a synthetic health claim to health-related action. The model explicitly couples system-level evidence visibility with user verification practices, incorporates verification burden and institutional responsibility, and organizes these relationships into five traceability functions, four user practices, calibrated trust, risk-proportionate safeguards, and testable propositions.

We argue that resilient use depends on the interaction between inspectable information systems and context-sensitive user practices. Origin, claim support, evidence limitations, production procedures, and correction histories become relevant because they inform decisions, not because disclosure is intrinsically protective. Table 1 identifies how this decision-oriented architecture complements adjacent frameworks without claiming that those frameworks exclude related concerns.

**Table 1:** Relationship between the proposed model and adjacent frameworks.

Framework or literature	Primary object of evaluation	Main contribution	Complementary emphasis in the present integration
Integrated health literacy model (15)	Person interacting with health information	Access, understand, appraise, and apply	Operationalizes capabilities through inspectable claim-to-decision evidence pathways and verification effort.
eHealth and digital health literacy (16, 17)	User skills in networked environments	Search, evaluate, communicate, and protect privacy	Connects online appraisal capabilities to specific system traceability functions and shared verification responsibilities.
RECAP for consumer generative AI (19)	Quality of an AI health response	Relevance, Evidence-based, Clarity, Adaptability, and Precision; clinical safety is a cross-cutting concern.	Connects answer-level appraisal dimensions to claim support, correction records, and appropriate reliance.
AI Health Literacy (18)	Reflective judgment about LLM use	Task appropriateness, context of use, and critical verifiability	Specifies complementary evidence-pathway functions and measurable verification burden alongside task and context competencies.
Multilevel susceptibility review (20)	Vulnerability to digital health misinformation	Individual, relational, identity, and platform factors	Organizes susceptibility conditions around evidence inspection and decision practices without replacing the multilevel account.
Multilayered epistemic disruption (21)	Mechanisms of AI-driven health misinformation	Discursive, biometric, temporal, and systemic disruption	Makes the decision pathway and verification burden explicit while treating epistemic dependence as a hypothesis.
Stakeholder AI health literacy	Patient, clinician, and governance competencies (22)	Trust calibration, critical appraisal, and responsible implementation	Decision-oriented traceability functions and verification effort complement this stakeholder organization.

Provenance concerns content origin, history, transformation, and custody. Transparency is the broader disclosure of information about a system or process; disclosure may not be usable for verification. Source traceability is used here as an action-oriented construct: the ability to follow a consequential claim through an inspectable pathway to origin, supporting evidence, procedural context, and relevant corrections. Claim traceability maps an assertion to offered sources; evidentiary traceability exposes the information needed to appraise their adequacy; procedural traceability records generation, retrieval, review, and transformation. Auditability concerns retrospective examination of process or system records and is broader and often institution-oriented. Explainability concerns how or why an output was produced, not necessarily its evidence. Evidence quality is the substantive strength and decision-relevance of support. Traceability is therefore neither a synonym for transparency nor a guarantee of truth. These distinctions guide the model rather than posit disjoint categories (23, 24).

## 2 Evidence base and analytic approach

This Conceptual Analysis uses a targeted integrative approach, not a systematic review. The original evidence base was assembled through September 2, 2026 using PubMed-indexed literature, Crossref and OpenAlex metadata, and official WHO, NIST, and C2PA resources. Search combinations linked generative AI or large language models with health misinformation, digital/eHealth literacy, trust, source transparency, provenance, citation fabrication, correction, and infodemic management. Targeted revision checks through September 15, 2026 evaluated the reviewer-suggested sources and adjacent literature on evidentiary dependence and AI governance. Selection is purposive and concept-driven; neither exhaustive coverage nor prevalence estimates are claimed.

Foundational health and digital health literacy models anchor the capability domain; recent empirical and conceptual sources supplement them with misinformation mechanisms, AI-specific failure modes, provenance functions, and institutional responses. A source was relevant if it defined a capability or system function, documented a health-information failure mode, clarified trust or verification, informed public health responses, or challenged the proposed model. Purely technical performance studies without a connection to user evaluation or health decisions, conceptually peripheral commentaries, and studies adding no distinct function were not priorities. This is an inclusion rationale for conceptual mapping, not a retrospective claim of comprehensive screening or enumerated exclusions.

Conceptual mapping compares the object of evaluation across these literatures: user capability, answer quality, source origin and history, evidentiary adequacy, susceptibility, correction, and accountability. Foundational models are compared with newer AI-literacy, misinformation, provenance, and governance approaches to check contribution boundaries and competing interpretations. Source and framework references inform these targeted checks, but no complete backward/forward citation-search ledger is claimed. Coverage is sufficient for the stated synthesis insofar as each proposed function and relevant competing construct has an identifiable conceptual basis. We do not claim qualitative saturation, a prospectively specified stopping rule, independent dual-author screening, or inter-rater reliability. The resulting organization is analytical and empirically testable, not evidence of established causal effects.

OpenAI Codex assisted with literature organization, drafting, language editing, bibliographic checks, and author-reviewable Python figure code. AI assistance is not a substitute for author source appraisal or responsibility. No human-participant data, empirical results, screening counts, or agreement statistics were generated for this article. The complete disclosure appears after the main text.

## 3 Why synthetic health information changes the credibility problem

### 3.1 Fluency can become an authority cue

Generative AI interfaces reduce the friction of health information seeking. They can translate terminology, organize questions, and adapt explanations to a user’s stated needs. This responsiveness may increase perceived relevance and interpersonal warmth. In one comparison of responses to patient questions, chatbot answers were often rated favorably for quality and empathy (25). Such findings identify a communication opportunity, but they do not establish factual reliability across conditions or justify autonomous clinical use.

The same properties that make an answer readable can become credibility cues. Coherence, specificity, confident tone, and apparent personalization can be mistaken for evidence quality. This risk is consistent with broader research showing that people may either resist medical AI or appreciate algorithmic advice depending on context and framing (26, 27). Trust is therefore not a fixed response to an AI label. It is shaped by interface cues, task stakes, perceived expertise, and whether users can evaluate the basis of a recommendation.

Synthetic fluency matters because it can conceal uncertainty. A conversational answer does not naturally display which statements are retrieved, inferred, generalized, or generated. It may combine strong evidence, weak evidence, and model completion within one uninterrupted paragraph. Readers then face a compression problem: several epistemically different claims arrive in the same rhetorical form. Without visible distinctions, confidence can be calibrated to presentation quality rather than evidentiary strength.

### 3.2 Citations can simulate traceability

A source list is not equivalent to source traceability. Earlier evaluations found fabricated citations and errors in authentic references (10–12). SourceCheckup subsequently tested whether the medical statements in LLM responses were supported by their cited sources; valid links did not ensure fully supported answers, including for retrieval-augmented models (13). A five-chatbot audit also evaluated accuracy, reference authenticity/completeness, and readability, but its adversarial prompts limit extrapolation to routine use (14). A health-specific systematic review situates such findings within heterogeneous evidence on generative production, propagation, and mitigation of misinformation (28). None validates the present framework.

This distinction reveals three different questions. Does the source exist? Does it support the specific claim? Is it adequate for the user’s context and intended action? Conventional citation display often answers only the first question, and sometimes not reliably. Claim-level mapping is needed for the second question. Source qualification, including study design, currency, population, and institutional authority, is needed for the third.

Citation simulation can be especially harmful in health contexts because users may lack database access, time, language proficiency, or domain knowledge. Verification becomes an additional task imposed after the interface has already delivered a convenient answer. When the verification burden is high, the visual presence of citations may operate as a stopping cue rather than an invitation to inspect evidence.

### 3.3 Synthetic corroboration can obscure independence

Corroboration requires evidentiary independence, not merely another phrasing. Apparently independent AI outputs may not provide independent confirmation because systems can rely on overlapping source ecosystems, retrieval infrastructures, or derivative content. This is a conceptually important possibility, also raised in accounts of a synthetic chorus (21), but direct empirical evidence on cross-system evidentiary dependence in consumer health contexts remains limited. Agreement between outputs should therefore prompt examination of their underlying sources rather than be treated as proof of independence.

Research on social bots demonstrates automated amplification of low-credibility social-media content (29); it does not establish dependence among LLM outputs. The practical distinction is between repeated presentation and independently generated evidence. Triangulation should compare accountable institutions, evidence types, and data-generating processes, while recognizing that institutional difference alone does not guarantee independent evidence.

### 3.4 Correction becomes a versioning problem

Misinformation can persist after correction because prior beliefs, identity commitments, and familiar explanations continue to influence reasoning (30). Synthetic answers add a versioning problem. An output may be corrected in one session while screenshots, copied text, derivative posts, or later model outputs continue to circulate. Users may not know that a claim changed, why it changed, or which version is current.

Correction should therefore be traceable. A useful system would preserve the time of generation, relevant knowledge cutoff or retrieval time, material updates, and the reason for correction. This function differs from simple source attribution. It supports contestability and helps users revise trust when evidence changes.

## 4 Digital health literacy is necessary but insufficient

### 4.1 From access and comprehension to critical verifiability

The integrated model of health literacy emphasizes access, understanding, appraisal, and application (15). The eHealth literacy model adds traditional, information, media, health, computer, and scientific literacies (16). Later instruments broaden digital health literacy to include searching, evaluating reliability, determining relevance, adding self-generated content, and protecting privacy (17). These capacities remain relevant to generative AI.

Generative systems nevertheless change the practical meaning of appraisal. Users are not always selecting among documents. They may be evaluating an answer that has synthesized or invented its apparent evidence base. Critical verifiability therefore becomes a joint property. The user needs skills to question and compare. The system needs features that make those actions possible.

AI Health Literacy emphasizes task appropriateness, context of use, and critical verifiability (18); the stakeholder-oriented framework also links literacy to governance (22). The present model complements these directions by specifying which evidence pathways must be inspectable, which decision practices they can support, and who bears the cost of verification. These are differences in organizing emphasis rather than claims that existing frameworks omit systems or accountability.

### 4.2 Confidence is not competence

Self-reported confidence should not substitute for observed appraisal performance. A systematic review associates lower health literacy with difficulties evaluating online health information (31), while multilevel work situates susceptibility within individual, relational, and platform conditions (20). Neither finding implies that confidence alone measures verification competence. In AI settings, assessments should examine actual appraisal and reliance as well as self-efficacy.

This gap matters because generative interfaces are easy to use. Conversational fluency can increase the feeling of successful information seeking without increasing the quality of verification. Literacy interventions should therefore assess behavior, including whether users open sources, detect claim-source mismatch, identify uncertainty, and seek professional advice when necessary. Knowledge tests and self-efficacy measures are insufficient on their own.

### 4.3 The burden of verification is unequally distributed

Digital literacy is increasingly understood as a determinant of health rather than a purely individual attribute (32). Access to devices, connectivity, language-concordant information, disability accommodations, time, and trusted care all shape whether a person can verify an AI answer. Recommendations for digital health equity likewise emphasize structural and organizational conditions (33).

Available or nominal traceability means that an evidence pathway technically exists. Usable or effective traceability means that the intended user can follow and understand it with effort proportionate to the decision. A system can expose links yet fail this test when verification requires excessive navigation, specialist knowledge, paywalled access, inaccessible language, or substantial time. Effective traceability therefore combines available pathways with accessibility, comprehensibility, and reasonable verification effort. Institutions should organize the evidence rather than transfer disproportionate labor to people with fewer resources.

## 5 A traceability-centered resilience model

The model connects information infrastructure, user appraisal, and institutional accountability (Figure 1). Calibrated trust means that confidence and action are proportionate to evidence quality, uncertainty, and potential harm. Its empirical target is discrimination and appropriate reliance, not uniformly high or low trust. Effective traceability is an affordance-in-use: evidence visibility contributes only when users can access and understand it at a reasonable verification cost.

**Figure 1:**
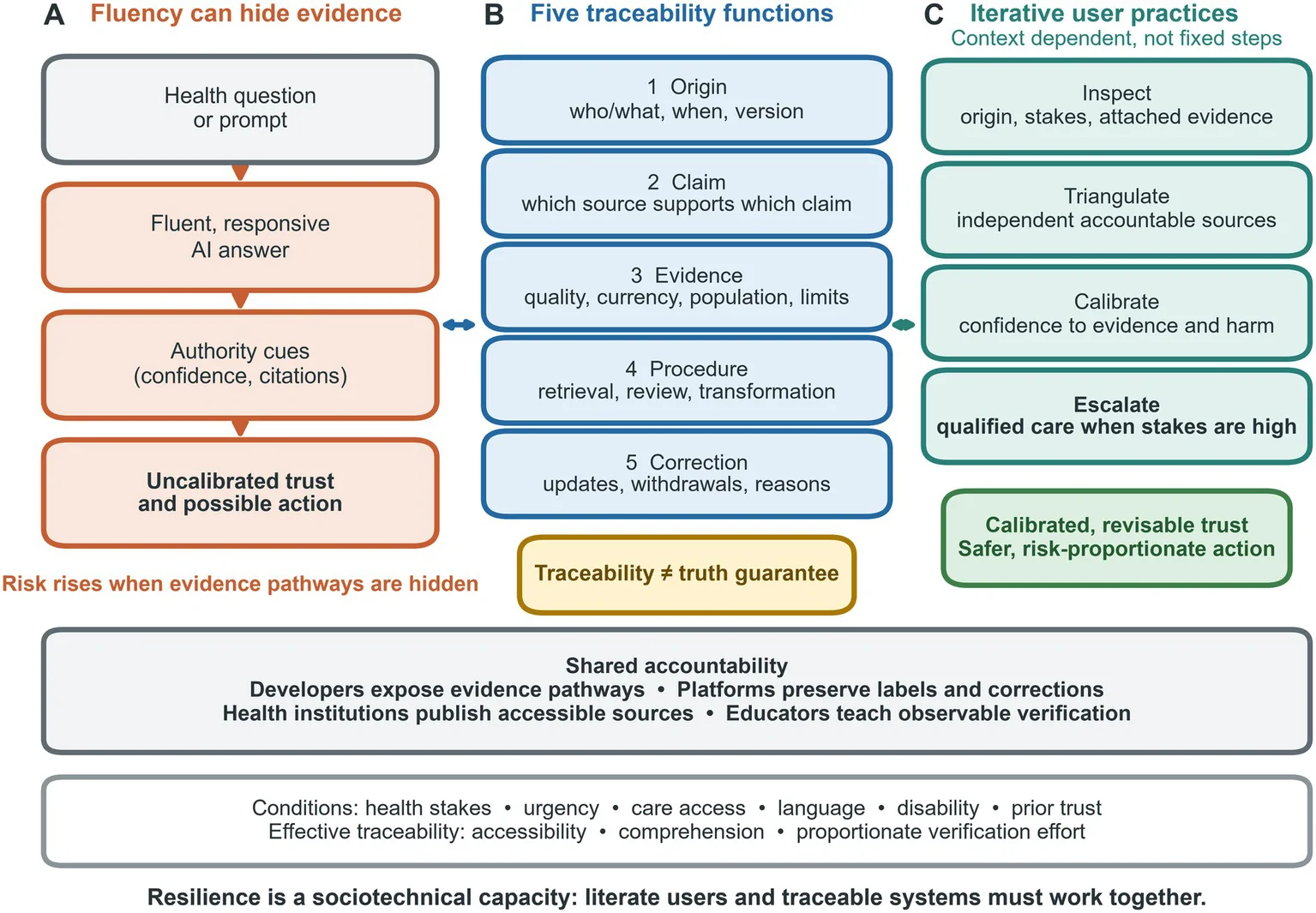
Traceability-centered resilience model for generative AI health information. **(A)** Fluent presentation and authority cues illustrate a possible route to uncalibrated trust when evidence is hidden, not an empirically established causal sequence. **(B)** Five complementary traceability functions make origin, claim support, evidence limits, procedures, and corrections inspectable. **(C)** Inspect, triangulate, calibrate, and escalate are iterative and context-dependent practices, not a mandatory algorithm; high-consequence situations may warrant immediate escalation. Bidirectional links indicate interacting sociotechnical layers rather than deterministic effects. Calibrated, revisable trust is a proposed outcome, not a guaranteed endpoint. Shared accountability supports accessible evidence pathways and proportionate verification effort. Consequence, access, language, disability, prior trust, and verification cost condition these relationships.

The five functions organize recurring gaps across literatures: provenance/authenticity motivates origin and history (23, 24); citation evaluation motivates claim-source alignment (10–13); health-information appraisal motivates evidence adequacy (15, 17, 31); AI-risk documentation motivates procedural records (24); and misinformation correction motivates updates and versioning (30). The four practices translate these affordances into decision actions: inspect available support, compare independent evidence, calibrate reliance, and escalate when consequence exceeds available evidence or competence (15, 17, 18). This is an analytically derived, empirically testable organization, not a factor-analytically established taxonomy or validated causal model.

### 5.1 Five traceability functions

Origin traceability identifies who or what generated, retrieved, reviewed, or published an output and when this occurred. AI disclosure is part of origin traceability, but an AI label alone provides little evidence about accuracy.

Claim traceability maps a specific health claim to the source or sources offered in support. The mapping should be granular enough to separate factual statements, recommendations, risk estimates, and uncertainty statements.

Evidentiary traceability communicates why a source is relevant and where its limits lie. Useful fields include source type, publication date, population, study design, guideline status, and material conflicts or retractions. This function helps distinguish a source that merely exists from one that is adequate for the decision.

Procedural traceability records how the answer was produced. Relevant information can include whether retrieval was used, the retrieval date, the model version, important transformation steps, and whether a qualified human reviewed the output. This function supports auditing without requiring disclosure of proprietary model internals.

Correction traceability preserves material revisions, withdrawn claims, and reasons for change. It turns correction into an observable process and supports the revision of user confidence.

These functions are complementary, not mutually exclusive categories. C2PA can make origin and editing records tamper-evident, while NIST treats provenance tracking as one element of voluntary generative AI risk management (23, 24). Such records authenticate assertions about history rather than determine health-claim truth. Connecting them to claim-level evidence appraisal is the action-oriented extension proposed here.

### 5.2 Four literacy practices

The practices are functionally distinct but iterative and context dependent. Their readable presentation order is not a fixed linear sequence or mandatory four-step algorithm. Users may skip, repeat, or return to practices; high-consequence situations may warrant immediate escalation before extensive user-led verification. Figure 1 represents interacting functions and layers, not deterministic transitions.

#### 5.2.1 Inspect

The user identifies the task, the health stakes, the origin of the answer, and the evidence attached to consequential claims. Inspection includes recognizing when an answer offers no inspectable support.

#### 5.2.2 Triangulate

The user compares a consequential claim with accountable sources whose evidence is genuinely independent where possible. Independence concerns underlying studies, source ecosystems, and data-generating processes as well as institutional identity. Multiple AI summaries are not, by themselves, evidence of independent confirmation.

#### 5.2.3 Calibrate

The user adjusts confidence to source quality, agreement, uncertainty, and potential harm. Calibration can result in provisional acceptance, continued uncertainty, or rejection. It avoids both automation bias and reflexive algorithm aversion.

#### 5.2.4 Escalate

The user seeks a clinician, pharmacist, public health authority, or emergency service when a claim could change diagnosis, treatment, medication, urgent care, or another high-consequence action. Escalation is not a failure of literacy. It is an appropriate literacy practice when available evidence and user competence are insufficient for the stakes.

Future studies can operationalize calibrated trust through correspondence between subjective confidence and externally appraised accuracy or evidentiary adequacy; detection of unsupported claims; and acceptance of stronger outputs while withholding or rejecting weaker ones. Logged source opening, claim-source checking, and independent-source seeking capture verification behavior. Task-specific expert or guideline-based standards can assess whether intended action matches evidence and risk, including appropriate professional escalation. Reference standards, scoring rules, and consequences should be prespecified: source quality and factual accuracy are related but not interchangeable targets. Table 2 gives illustrative candidate indicators, not a validated measurement instrument.

**Table 2:** Illustrative operational indicators and candidate measures for the proposed model.

Construct	Example observable indicator	Possible empirical measure
Origin traceability	Generator, publisher, model, date, and version identifiable	Proportion of outputs with identifiable origin/version metadata
Claim traceability	Consequential assertions linked to retrievable support	Valid claim-link proportion; independently appraised claim-source alignment rate
Evidentiary traceability	Design, date, population, guideline status, and limitations visible	Metadata completeness and user-comprehension/usability scores
Procedural traceability	Retrieval/date, model version, transformation, and review status accessible	Proportion with accessible procedural records; retrieval success
Correction traceability	Changes, withdrawals, dates, and reasons visible	Accessible version history; correction-notice detection
Inspect	Sources opened and support examined	Observed source opening and claim-source checking
Triangulate	Underlying evidence compared across independent accountable sources	Independently appraised source-comparison behavior
Calibrate	Confidence revised according to evidence and consequence	Confidence–reference-standard correspondence; calibration error
Escalate	Qualified help sought when consequence warrants it	Appropriate-referral rate and delay in high-consequence scenarios
Calibrated trust	Stronger and weaker support discriminated; reliance proportionate	Unsupported-claim detection; appropriate reliance; decision appropriateness
Verification burden	Time, navigation, comprehension, and access barriers observable	Completion time/clicks; comprehension difficulty; paywall/language barriers

These indicators are illustrative candidates, not a validated instrument. Confidence calibration, evidentiary adequacy, and decision appropriateness require task-specific reference standards. Accessibility, comprehension, and verification effort should be assessed for the intended population.

### 5.3 Institutional and platform accountability

Individual action is only one layer of resilience. AI developers determine whether an interface exposes sources, uncertainty, model version, and correction history. Health institutions determine which authoritative content is available in machine-readable and multilingual forms. Platforms determine how generated content is labeled, ranked, shared, and corrected. Educators determine whether literacy programs teach observable verification practices.

Health-literate systems aim to facilitate navigation and use of information (34, 35). For generative AI, this principle supports usable routes from claims to evidence and appropriate human support. Algorithmovigilance offers a complementary governance perspective: incident reports should capture model versions and contextual factors, while updated documentation should communicate risks accessibly (36). These proposals inform accountability design but do not prove that a particular traceability feature improves decisions. Recent clinical governance commentary also places algorithmovigilance alongside accountability, drift, and incident reporting (37). These are governance topics, not evidence that the present framework is effective.

## 6 From model to intervention

### 6.1 Interface design

Interfaces should connect consequential claims to visible evidence without overwhelming users. A layered display can show a concise source indicator first and allow deeper inspection of claim-source alignment, study design, date, population, and limitations. Source clarity has been shown to affect judgments in digital health communication (38). Research on provenance-enabled media also suggests that provenance cues can influence trust and accuracy perceptions, although effects depend on design and interpretation (39).

Citation count should not serve as a quality signal. One directly relevant guideline may be more useful than many peripheral articles. Proposed interfaces would flag unsupported claims, source disagreement, and material guideline changes, and open links at relevant passages where feasible. Evaluation should compare nominal availability with effective traceability, measuring time, navigation, comprehension, and access barriers as well as evidentiary discrimination.

Procedural information should be proportionate and usable. A raw model card or technical log will not help most users at the moment of decision. Interfaces can translate procedural traceability into concise statements such as “generated with retrieval from these sources on this date,” “not reviewed by a clinician,” or “this answer changed after a source update”.

### 6.2 Literacy education

Digital health literacy programs should teach verification as context-dependent, repeatable practices. Learners can identify consequential claims, open evidence, test claim-source alignment, compare independent authorities, and explain confidence revisions. A reverse-image-search intervention increased intention to use the tool but did not improve credibility judgments or misinformation discernment (40). Accuracy prompts improved the quality of sharing (41), and inoculation improved recognition of manipulation and related outcomes (42). These findings support testing targeted interventions, not assuming transfer to conversational health synthesis. A recent primary-care trial reported improved digital vaccine literacy and reduced parental hesitancy after structured education (43); it did not test AI traceability or establish which program component produced the changes.

Assessment should use performance tasks. A learner might compare a fluent answer with its cited guideline, detect a population mismatch, or decide when medication advice requires escalation. Measures should capture source opening, claim-source matching, confidence calibration, and appropriate referral. They should also test whether an intervention creates indiscriminate distrust, which would undermine legitimate digital health support.

### 6.3 Health institutions and public health agencies

Public health agencies should publish content that is authoritative, current, accessible, and technically retrievable. Structured claims, stable identifiers, update histories, plain-language versions, and multilingual resources can improve the evidence available to both users and retrieval-enabled AI systems. WHO identifies health and digital literacy, social listening, and access to reliable information as components of infodemic management (44).

Institutions should also develop correction pathways for synthetic content. These can include rapid reporting mechanisms, machine-readable advisories, versioned guidance, and partnerships with platforms. The objective is not to certify every AI answer. It is to reduce the time between detecting a harmful claim and updating the information environments that reproduce it.

### 6.4 Proposed risk-tiered safeguards

Potential harm can guide the intensity of proposed safeguards: general education differs from medication changes or urgent triage. Table 3 presents author-proposed, provisional conceptual decision aids. They are not validated clinical risk classifications, regulatory standards, or mandatory requirements. The tiers concern the consequences of intended action, not medical specialties; context can move a use case between tiers. Existing literacy and AI-risk guidance supports attention to task, context, and accountability (18, 24), whereas the particular safeguards and thresholds below are proposals requiring evaluation.

**Table 3:** Proposed risk-tiered traceability safeguards and user practices for generative AI health information.

Consequence category	Illustrative use	Proposed system safeguards	Proposed user practices	Proposed use boundary
Low consequence	General definitions, preparing questions for a visit	AI disclosure, source list, date, uncertainty statement	Inspect origin; compare at least one authoritative source when acting	No individualized diagnosis or treatment claim
Moderate consequence	Self-management, prevention choices, interpretation of nonurgent symptoms	Claim-level sources, source qualification, conflict display, update date	Triangulate independent sources; calibrate confidence; document uncertainties	Encourage professional confirmation when advice could materially change care
High consequence	Medication, diagnosis, emergency triage, pregnancy, severe symptoms	Verified current guidance, explicit limitations, audit log, human review or direct referral	Do not act on AI output alone; escalate to qualified care	Clear emergency and professional-care pathway; AI must not present itself as final authority

Author-proposed conceptual decision aid; not a validated clinical classification or regulatory standard. Proposed safeguards require empirical evaluation and adaptation to the intended use and setting.

## 7 Testable propositions and research agenda

Future evaluations should prioritize a recurring outcome set: evidentiary discrimination, confidence calibration, decision appropriateness, verification behavior, verification burden, and escalation behavior. Table 2 illustrates candidate indicators; operational definitions and reference standards must be specified for each study. Trust ratings alone cannot distinguish justified acceptance from over-reliance. The following propositions are hypotheses, not demonstrated effects.

Proposition 1: Claim-level traceability will improve confidence calibration more than generic source lists. Experiments can compare absent citations, bibliographies, sentence-level links, and evidence panels using calibration error, evidentiary discrimination, and decision appropriateness.

Proposition 2: Benefits will vary non-linearly with consequence level. Additional evidence may offer limited benefit for definitions and greater benefit for moderate-consequence decisions; high-consequence cases may require professional review or immediate escalation. Outcomes should include decision appropriateness and escalation behavior; neither the tiers nor these safeguards are validated thresholds.

Proposition 3: Source qualification will improve evidentiary discrimination when authentic citations are misaligned or inadequate. Manipulating existence, claim alignment, study design, currency, and population fit independently would separate bibliographic validity from evidentiary adequacy.

Proposition 4: Literacy instruction and usable interface traceability will interact to improve verification behavior and confidence calibration. Factorial designs can test additive or synergistic gains while checking for indiscriminate distrust.

Proposition 5: Verification burden will moderate whether available traceability becomes effective traceability and whether it reduces or widens inequities. Studies should measure time, clicks, comprehension, and access barriers, and stratify the core outcomes by language, disability, education, health literacy, access to care, and AI experience.

Proposition 6: Correction traceability will support proportionate confidence revision and appropriate reliance, conditional on institutional trust. Longitudinal comparisons of silent updates, visible notices, and evidence-linked explanations should assess revision, evidentiary discrimination, and continued reliance rather than willingness to reuse alone.

Future research should also examine adversarial conditions. Malicious actors can fabricate provenance, overwhelm users with low-quality citations, or exploit the appearance of institutional review. Studies should test how users interpret missing provenance, contested sources, and authentic provenance attached to misleading content. The relevant endpoint is not maximal trust in labeled content. It is better discrimination between claims that warrant action, caution, and rejection.

## 8 Equity, ethics, and governance

Traceability introduces ethical tradeoffs. More detailed origin records may expose creators, patients, or sensitive contexts. Verification logs can reveal health interests. Human review labels can create new authority cues without guaranteeing review quality. Provenance infrastructures may privilege institutions that can afford technical compliance while marginalizing community knowledge.

These risks require data minimization, privacy protection, accessible design, and contestability. Origin disclosure should identify accountable entities without exposing unnecessary personal data. Claim-level evidence should include community-relevant and local guidance when appropriate, while preserving standards for methodological quality. Correction systems should permit challenges and document institutional errors.

Governance should also address responsibility gaps. Developers should not describe a source feature as safety assurance when the system does not test claim-source alignment. Health institutions should not outsource public communication accountability to AI vendors. Platforms should not use an AI label as a substitute for reducing amplification of harmful content. Users retain agency, but system designers retain duties to make safe action realistically possible.

Human–AI combinations do not automatically outperform the better of humans or AI alone (45). Proposed high-consequence safeguards should therefore specify reviewer qualifications, the scope of review, and referral responsibilities rather than rely on a generic human-in-the-loop label. Algorithmovigilance also emphasizes post-deployment contextual monitoring and communication of adverse events (36). These are governance design principles to test; their applicability as legal duties depends on jurisdiction, intended use, and device status, which this article does not determine. In their Comment on non-certified generative AI, Sirago and colleagues distinguish documentation assistance from outputs that influence clinical decisions and propose controls adapted to intended use and workflow (46). This offers a clinical boundary comparison, not a regulatory classification of public-facing health information tools.

## 9 Limitations

This article offers a conceptual synthesis rather than a systematic review. The targeted search may omit relevant studies, and the rapidly changing evidence base limits claims about specific models or interface features. Several cited evaluations use earlier model versions, so their numerical error rates should not be generalized to all current systems. They remain evidence that fluency and citation display cannot be treated as proof of reliability.

The proposed traceability functions have not been validated as a unified model. Their effects may vary by culture, language, health system, clinical condition, and platform. Traceability can also be manipulated or misunderstood. Authentic provenance does not establish factual truth, and transparent uncertainty may reduce trust without improving decisions if users lack viable alternatives.

The risk tiers, operational indicators, and unified model require empirical validation. The indicators are examples, not a validated instrument; decision appropriateness and evidentiary adequacy require task-specific reference standards. The search was concept-driven rather than exhaustive and does not reconstruct an independent screening process or claim formal saturation. Verification burden and performance should be evaluated with underserved populations from the start, not generalized from digitally confident samples.

## 10 Discussion

The analysis locates a credibility problem at the transition from generated claim to health action. Its contribution is a decision-oriented integration of established literacy, provenance, verification, and accountability concepts, with functions and practices specified as testable targets. It does not propose a new literacy construct or establish the causal efficacy of the architecture. Alternative explanations, including source quality, prior knowledge, social identity, and access to trusted care, could account for apparent benefits of an intervention and should be separated experimentally.

Literacy and trust should be examined together without equating either with warranted reliance. The primary-care education trial provides complementary evidence that digital vaccine literacy and hesitancy can change within the same intervention (43), but does not demonstrate mediation or transfer to AI use. Multilevel susceptibility research also identifies relational trust as part of the information environment (20), and source-clarity experiments link identifiable and socially close senders to intended compliance (38). These findings motivate distinguishing generalized trust or mistrust from calibrated trust: the latter concerns differential reliance on stronger and weaker evidence and action proportionate to consequence. A cross-sectional primary-care study of 568 adults in Türkiye found medical mistrust and health system distrust associated with self-reported healthcare delay and less favorable cancer screening attitudes (47). These associations neither establish causation nor test AI-mediated information or traceability interventions; they provide contextual evidence that generalized mistrust and health-related responses should be examined separately from calibrated reliance.

Effective traceability can fail even when technically rich records are available. A link, provenance credential, or disclosure document is protective only insofar as it supports an intelligible decision at a reasonable cost. This shifts evaluation from disclosure volume to evidentiary discrimination and decision appropriateness, while treating access barriers and comprehension as model components. A system that raises average performance but increases the verification burden for underserved users would not satisfy the model’s equity objective.

Institutional accountability connects these decision and equity concerns to governance. Version-aware incident reporting and accessible risk documentation are consistent with algorithmovigilance proposals (36), while the present risk tiers specify provisional design hypotheses. Human review and referral are not asserted here to be universally effective or legally mandatory. Empirical evaluation must test their benefit, resource demands, and unintended authority effects in specified settings; applicable regulation requires a separate jurisdiction-specific assessment. The non-certified generative AI Comment proposes source attribution, versioning, logging, named responsibilities, and monitoring within clinical workflows (46). We use this as a normative comparison: public health information interfaces require their own user-centred evaluation, and clinical governance proposals cannot establish the efficacy or legal necessity of our safeguards.

## 11 Conclusion

Synthetic health information can attract trust without a usable route to adequate evidence. The proposed architecture connects five traceability functions with four iterative literacy practices and shared responsibility for verification effort. Its practical aim is calibrated trust and proportionate action, measured through discrimination, reliance, decision quality, and appropriate escalation. The framework and risk-tiered safeguards remain empirical proposals. Their value will depend on whether they improve decisions across real-world populations without shifting excessive costs to those least able to verify.
